# Case of Double Ovarian Dropsy

**Published:** 1851-06

**Authors:** E. R. Peaslee

**Affiliations:** Prof. of Surgery in the Medical School of Maine


*Case of double Ovarian Dropsy,— both Ovaries successfully removed by the large peritoneal section.* By E. R. Peaslee, A. M., M. D. Prof. of Surgery in the Medical School of Maine.

Professor Peaslee reports, in the American Journal of Medical Sciences, for April, 1851, a case in which both ovaries were successfully removed by the large peritoneal section. The length of the article precludes our inserting it in full. The tumors removed are thus described:

First. The large mass first removed now resembles, as a whole, in form and relation of parts (it being collapsed,) the fœtal membranes enclosing the placenta — the more solid part corresponding to the latter. Its vertical diameter is twelve inches, its transverse nine and a half inches; its original form being ovoid. The enclosed portion is oval and thin; being six and a half inches by four and a half inches in diameter, and two and a half inches thick.

The external surface of the *sac* is perfectly smooth and nacreous in appearance, except two inches square on the right side, and a little less on the left, (precisely where the "friction feeling" was perceived) — these surfaces being covered by the recently formed bands I had ruptured in breaking up the adhesions of the sac. An epithelium, but no distinct serous membrane can be detected by one of Chevallier's largest miscroscopes. The Fallopian tube, three-sixteenths of an inch in diameter, extends to the top of the solid tumor, accompanied by a large vein, and an artery of the size of a wheat straw. The protuberance on the solid portion, felt through the abdominal walls, was a distinct sac, as was supposed; its fluid having a specific gravity of 1023, and containing, like that of the large sac, a large quantity of albumen, and scales of cholesterine. The sac itself is three-sixteenths of an inch thick.

Its *internal* surface presents several large veins; and is covered quite extensively in patches by recently exuded plasma. In general appearance it resembles a mucous membrane.

The weight of the whole mass is two pounds, three ounces avoirdupois.

Second. The *right* ovarian tumor is one and a half inch long, by one and a fourth inch in diameter, consisting of a sac containing ℥iv of a reddish fluid; and the proper stroma of the organ, hypertrophied and indurated, and presenting a lobulated and warty appearance. Its whole weight is ʒx.

The following extract contains a summary of the symptoms and progress of the case after the operation:

"So far as the writer is aware, Miss G.——'s case is *unique*, as far as the successful removal of both ovaries at the same time by the peritoneal section is concerned. It is also scarcely less singular, for the very slight disturbance of any kind from the operation. The *pulse* never rose above 120; never above 115 after the first twenty-fours had elapsed, except for a few minutes on Sunday night. From Monday morning to Tuesday morning, it ranged between 93 and 114; Tuesday to Wednesday morning, between 86 and 112; Wednesday to *Friday* morning, between 88 and 104; Friday to Saturday morning, 83 and 100. It never rose subsequently above 97, and averaged 90 — about her natural pulse. The *respiration* was 26 for half an hour when reaction was first established; and uniformly 23 or 24 during the first three days. It then continued at 22, till the pulse fell to 90; and then became 20. The *reaction* also was perfect in less than five hours, and was never excessive. Indeed, the patient may almost be said to have recovered without a single bad symptom; at least without any severe symptoms peculiar to such an operation; or which might not have occurred to one of her delicacy of constitution, from even a slight cause. Hence the *medicinal* part of the after treatment was decidedly expectant. The suppuration could not have amounted to more than ℥iss in all, during recovery.

We give the concluding remarks of the distinguished operator:

"The success of the operation I attribute to the fortitude and confidence of the patient; the comparatively slight adhesion of the diseased mass; the temperature, &c., of the room at the time and subsequently; accurate coaptation of the divided abdominal walls; and the judicious after-treatment of Dr. Jarvis, seconded by the faithful attentions of the three young gentlemen before named. I am positive that as much care and skill are necessary in closing the incision properly, as in performing the preceding operation.

Whether the operation of ovariotomy is ever justifiable, is a question which would certainly be out of place here. It is the writer's opinion that, if the patient's general health is rapidly failing (but not already too far prostrated,) and the tumor is found to be not extensively adherent, so far as all the known methods, taken *together*, can decide that question, the operation *is* justifiable; *provided* the patient, after fully understanding its nature, strongly desires to have it performed, and has strong hopes of recovery therefrom. But it is an operation never to be urged, nor to be undertaken by an operator whose care does not include the minutest particulars, both prior and subsequent to its performance, which can affect its results.

And I cannot close without alluding to the obligations under which the medical profession in our country has been placed, by the full and precise reports of his now numerous operations for the removal of ovarian diseases, which Dr. W. L. Atlee has given from time to time in this Journal. But for these, my patient might perhaps not have been rescued from an early death. For only accurate and minute reports of such cases are of any practical value to others; and this is the writer's apology for the length of this communication.

DART. COLLEGE, *Feb.* 1851.

